# Survival benefit of glioblastoma patients after FDA approval of temozolomide concomitant with radiation and bevacizumab: A population-based study

**DOI:** 10.18632/oncotarget.17054

**Published:** 2017-04-12

**Authors:** Ping Zhu, Xianglin L. Du, Guangrong Lu, Jay-Jiguang Zhu

**Affiliations:** ^1^ Department of Epidemiology, Human Genetics, and Environmental Sciences, The University of Texas Health Science Center at Houston (UTHealth), School of Public Health, Houston, TX 77030, USA; ^2^ The Vivian L. Smith Department of Neurosurgery, The University of Texas Health Science Center at Houston (UTHealth), McGovern Medical School, and Memorial Hermann at Texas Medical Center, Houston, TX 77030, USA

**Keywords:** glioblastoma (GBM), temozolomide, bevacizumab, overall survival, cancer registry

## Abstract

Few population-based analyses have investigated survival change in glioblastoma multiforme (GBM) patients treated with concomitant radiotherapy-temozolomide (RT-TMZ) and adjuvant temozolomide (TMZ) and then bevacizumab (BEV) after Food and Drug Administration (FDA) approval, respectively. We aimed to explore the effects on survival with RT-TMZ, adjuvant TMZ and BEV in general GBM population based on the Surveillance, Epidemiology, and End Results (SEER) and Texas Cancer Registry (TCR) databases. A total of 28933 GBM patients from SEER (N = 24578) and TCR (N = 4355) between January 2000 and December 2013 were included. Patients were grouped into three calendar periods based on date of diagnosis: pre-RT-TMZ and pre-BEV (1/2000–2/2005, P1), post-RT-TMZ and pre-BEV (3/2005–4/2009, P2), and post-RT-TMZ and post-BEV (5/2009–12/2013, P3). The association between calendar period of diagnosis and survival was analyzed in SEER and TCR, separately, by the Kaplan-Meier method and Cox proportional hazards model. We found a significant increase in median overall survival (OS) across the three periods in both populations. In multivariate models, the risk of death was significantly reduced during P2 and further decreased in P3, which remained unchanged after stratification. Comparison and validation analysis were performed in the combined dataset, and consistent results were observed. We conclude that the OS of GBM patients in a “real-world” setting has been steadily improved from January 2000 to December 2013, which likely resulted from the administrations of TMZ concomitant with RT and adjuvant TMZ for newly diagnosed GBM and then BEV for recurrent GBM after respective FDA approval.

## INTRODUCTION

Glioblastoma multiforme (GBM) comprises approximately 46.1% of primary malignant brain tumors, and only about 5.1% of patients survive five years after diagnosis [1]. Favorable clinical prognostic factors include maximum safe resection, good performance status, young age at diagnosis, completion of radiation and chemotherapies [1], [2], [3], [4]. Molecular prognostic factors include the presence of the O^6^-methylguanine-DNA methyltransferase (*MGMT*) promoter methylation [5], [6], the isocitrate dehydrogenase 1 and 2 (*IDH1/2*) mutations [7]. Besides the known hyper-methylation of the MGMT gene, Noushmehr et al [8] revealed that glioma cytosine–phosphate–guanine islands methylator phenotype (G-CIMP) is a positive prognostic marker. Furthermore, two studies have categorized GBM into 3 subtypes [mesenchymal (Mes), proneural (PN) and proliferative] [9], or 4 subtypes (Mes, PN, neuronal and classical) [10], respectively. Among all the subtypes of GBM, Phillips et al [9] found that PN GBM patients has the best prognosis.

Stupp and colleagues from the European Organization for Research and Treatment of Cancer (EORTC) Brain Tumor and Radiotherapy Groups and the National Cancer Institute of Canada (NCIC) Clinical Trials Group demonstrated that median overall survival (OS) of GBM patients improved to 14.6 months with concurrent radiation therapy and temozolomide (RT-TMZ) followed by TMZ alone for 6 cycles in a phase III randomized controlled trial (RCT) published in March 2005 [11]. On March 15, 2005, the U.S. Food and Drug Administration (FDA) approved TMZ, an oral alkylating agent, in concurrent usage with RT followed by maintenance treatment with TMZ as a new standard of care (SOC) for newly diagnosed adult GBM [12]. For patients with recurrent GBM prior to FDA approval of bevacizumab (BEV), various therapies had been applied including second craniotomy with or without carmustine implantation (Gliadel wafers), salvage radiation, dose-dense TMZ, nitrosoureas, carboplatin, PCV (procarbazine, lomustine and vincristine), etoposide or irinotecan [13], [14], [15], [16]. Based on the results of two phase II trials by Friedman et al (AVF3708g) [17] and Kreisl at al (National Cancer Institute 06-C-0064E) [18], BEV, a humanized monoclonal antibody against the vascular endothelial growth factor (VEGF), received accelerated approval for recurrent GBM therapy by FDA on May 9, 2009 [19].

It is known that GBM patients enrolled in RCTs have better prognosis and longer survival than those who were not enrolled in clinical trials [20]. Participants in clinical trials need to pass stringent selection criteria, including good performance status, satisfactory laboratory parameters, minimal comorbidity, normal organ status, and adequate bone marrow function [11], [17], [18]. Therefore, the impacts of RT-TMZ followed by TMZ and subsequently BEV for recurrent GBM on survival in a “‘real-world’ setting” should be further investigated. Four population-based studies concluded that the usage of TMZ [21], [22], [23], [24] was associated with improved GBM survival based on the Surveillance, Epidemiology, and End Results (SEER) Program database. The time periods of GBM diagnosis studied in these four studies were 1993-2007, 2000-2006, 2001-2007 and 2000-2008, respectively. One limitation of the SEER database is the absence of individual patient's chemotherapy information. Dubrow et al [25] demonstrated that application of TMZ can fully explain the improved OS among GBM patients diagnosed between 1997 and 2008 from the Veterans Health Administration (VHA) dataset which had the access to individual chemotherapy data.

The impact of BEV on progression-free survival (PFS) and OS for newly diagnosed GBM has been investigated by two separate phase III RCTs. Chinot et al (AVAglio) [26] and Gilbert et al (RTOG 0825) [27] demonstrated that application of BEV during RT-TMZ period does not improve OS in newly diagnosed GBM patients compared to the control arms, independently. But they cannot conclude whether BEV therapy was beneficial to GBM patients or not overall, since patients from the control arms received BEV as well after GBM progression. Two population-based studies reported positive impact of BEV on OS of GBM patients by utilizing the SEER database [28], [29]. But there are limitations in those two studies: 1) Both of them performed analyses with limited follow up durations; 2) The usage of BEV was estimated based on three years of death records (2006, 2008 and 2010) from Johnson et al report [28]; 3) A limited number of GBM patients diagnosed between 2000 and 2009 was studied in Wachtel et al study [29]. In our opinion, they may not achieve a meaningful comparison of OS between pre-and post-BEV eras since BEV was approved by FDA on May 9, 2009. Therefore, further investigations related to the role of BEV on survival in “real-world” GBM population are needed.

In the present study, we constructed a retrospective cohort of patients diagnosed with GBM between January 2000 and December 2013 derived from the SEER, TCR and a pooled data from both SEER and TCR. We aim to test the hypotheses that the administration of TMZ and BEV after FDA approval, respectively, would explain the improved survival in GBM patients across three calendar periods of diagnosis, and the findings in SEER, TCR and the combined dataset would be consistent.

## RESULTS

### Patients demographic and clinical characteristics in SEER and TCR datasets

Table 1 shows the demographic and clinical features of grouped GBM patients at diagnosis. A total of 24578 GBM patients were extracted from the SEER dataset, and 21364 (86.9%) of them died during the observation period between January 2000 and December 2013. From the TCR database, 3779 (86.8%) GBM patients deceased within the same period in a total of 4355 patients identified. The majority of patients were more than 50 years old at diagnosis with mean±standard deviation (SD): SEER, 61.2±12.9 years; TCR, 60.6±13.0 years, respectively, and with male (SEER: 58.7%, TCR: 59.9%) and Caucasian (SEER: 80.4%, TCR: 75.2%) predominate.

**Table 1:** Baseline characteristics of GBM patients from SEER, TCR and the combined dataset, by calendar period of diagnosis

	Jan 2000-Feb 2005 (P1)				Mar 2005-Apr 2009 (P2)				May 2009-Dec 2013 (P3)				Total (P1, P2 and P3)				*P^e^*	*P^f^*
	SEER	TCR	Combined	*P^a^*	SEER	TCR	Combined	*P^b^*	SEER	TCR	Combined	*P^c^*	SEER	TCR	Combined	*P^d^*		
Characteristics	N=8169	N=1357	N=9526		N=7420	N=1286	N=8706		N=8989	N=1712	N=10701		N=24578	N=4355	N=28933			
Age at diagnosis, N (%)								* *										
20-49	1660 (20.3)	273 (20.1)	1933 (20.3)	<0.001	1310 (17.7)	238 (18.5)	1548 (17.8)	0.832	1409 (15.7)	290 (16.9)	1699 (15.9)	0.231	4379 (17.8)	801 (18.4)	5180 (17.9)	0.008	<0.001	0.001
50-59	2004 (24.5)	399 (29.4)	2403 (25.2)		2010 (27.1)	341 (26.5)	2351 (27.0)		2270 (25.3)	433 (25.3)	2703 (25.3)		6284 (25.6)	1173 (26.9)	7457 (25.8)			
60-69	2097 (25.7)	359 (26.5)	2456 (25.8)		2091 (28.2)	353 (27.5)	2444 (28.1)		2852 (31.7)	558 (32.6)	3410 (31.9)		7040 (28.6)	1270 (29.2)	8310 (28.7)			
70-	2408 (29.5)	326 (24.0)	2734 (28.7)		2009 (27.1)	354 (27.5)	2363 (27.1)		2458 (27.3)	431 (25.2)	2889 (27.0)		6875 (28.0)	1111 (25.5)	7986 (27.6)			
Sex, N (%)																		
Male	4825 (59.1)	818 (60.3)	5643 (59.2)	0.399	4388 (59.1)	784 (61.0)	5172 (59.4)	0.218	5219 (58.1)	1005 (58.7)	6224 (58.2)	0.621	14432 (58.7)	2607 (59.9)	17039 (58.9)	0.158	0.279	0.426
Female	3344 (40.9)	539 (39.7)	3883 (40.8)		3032 (40.9)	502 (39.0)	3534 (40.6)		3770 (41.9)	707 (41.3)	4477 (41.8)		10146 (41.3)	1748 (40.1)	11894 (41.1)			
Race/Ethnicity, N (%)																		
White	6735 (82.4)	1074 (79.1)	7809 (82.0)	<0.001	5957 (80.3)	980 (76.2)	6937 (79.7)	<0.001	7076 (78.7)	1220 (71.3)	8296 (77.5)	<0.001	19768 (80.4)	3274 (75.2)	23042 (79.6)	<0.001	<0.001	<0.001
Black	415 (5.1)	72 (5.3)	487 (5.1)		394 (5.3)	87 (6.8)	481 (5.5)		532 (5.9)	110 (6.4)	642 (6.0)		1341 (5.5)	269 (6.2)	1610 (5.6)			
Hispanic	697 (8.5)	196 (14.4)	893 (9.4)		747 (10.1)	197 (15.3)	944 (10.8)		900 (10.0)	322 (18.8)	1222 (11.4)		2344 (9.5)	715 (16.4)	3059 (10.6)			
Others	322 (3.9)	15 (1.1)	337 (3.5)		322 (4.3)	22 (1.7)	344 (4.0)		481 (5.4)	60 (3.5)	541 (5.1)		1125 (4.6)	97 (2.2)	1222 (4.2)			
Marital status, N (%)																		
Single	953 (11.7)	173 (12.8)	1126 (11.8)	0.021	963 (13.0)	164 (12.8)	1127 (12.9)	0.119	1343 (14.9)	272 (15.9)	1615 (15.1)	0.261	3259 (13.3)	609 (14.0)	3868 (13.4)	0.001	<0.001	0.068
Married	5582 (68.3)	955 (70.4)	6537 (68.6)		5062 (68.2)	910 (70.8)	5972 (68.6)		6027 (67.1)	1157 (67.6)	7184 (67.1)		16671 (67.8)	3022 (69.4)	19693 (68.1)			
DWS	1634 (20.0)	229 (16.9)	1863 (19.6)		1395 (18.8)	212 (16.5)	1607 (18.5)		1619 (18.0)	283 (16.5)	1902 (17.8)		4648 (18.9)	724 (16.6)	5372 (18.6)			
Tumor site, N (%)																		
Supratentorial	6137 (75.1)	1072 (79.0)	7209 (75.7)	0.002	5832 (78.6)	1023 (79.6)	6855 (78.7)	0.442	7036 (78.3)	1328 (77.6)	8364 (78.2)	0.519	19005 (77.3)	3423 (78.6)	22428 (77.5)	0.063	<0.001	0.388
Infratentorial/NOS	2032 (24.9)	285 (21.0)	2317 (24.3)		1588 (21.4)	263 (20.5)	1851 (21.3)		1953 (21.7)	384 (22.4)	2337 (21.8)		5573 (22.7)	932 (21.4)	6505 (22.5)			
Surgery, N (%)																		
No surgery	1683 (20.6)	244 (18.0)	1927 (20.2)	0.001	1475 (19.9)	216 (16.8)	1691 (19.4)	0.007	1438 (16.0)	293 (17.1)	1731 (16.2)	0.021	4596 (18.7)	753 (17.3)	5349 (18.5)	<0.001	<0.001	0.315
Local excision/biopsy	1499 (18.4)	288 (21.2)	1787 (18.8)		1433 (19.3)	260 (20.2)	1693 (19.4)		1940 (21.6)	329 (19.2)	2269 (21.2)		4872 (19.8)	877 (20.1)	5749 (19.9)			
Partial resection	2320 (28.4)	344 (25.4)	2664 (28.0)		2035 (27.4)	330 (25.7)	2365 (27.2)		2718 (30.2)	490 (28.6)	3208 (30.0)		7073 (28.8)	1164 (26.7)	8237 (28.5)			
GTR	2667 (32.7)	481 (35.5)	3148 (33.0)		2477 (33.4)	480 (37.3)	2957 (34.0)		2893 (32.2)	600 (35.1)	3493 (32.6)		8037 (32.7)	1561 (35.8)	9598 (33.2)			
Radiation, N (%)																		
Untreated	1659 (20.3)	261 (19.2)	1920 (20.2)	0.361	1425 (19.2)	388 (30.2)	1813 (20.8)	<0.001	1565 (17.4)	683 (39.9)	2248 (21.0)	<0.001	4649 (18.9)	1332 (30.6)	5981 (20.7)	<0.001	<0.001	<0.001
Treated	6510 (79.7)	1096 (80.8)	7606 (79.8)		5995 (80.8)	898 (69.8)	6893 (79.2)		7424 (82.6)	1029 (60.1)	8453 (79.0)		19929 (81.1)	3023 (69.4)	22952 (79.3)			

Abbreviation: DWS, divorced or widowed or separated; NOS, not otherwise specified; GTR, gross total resection.

a, b, c and d: Pearson's χ2 test was conducted to compare the proportions of baseline characteristics between SEER and TCR within P1, P2 and P3, respectively.

e and f: Pearson's χ2 test was conducted to compare the proportions of baseline characteristics across calendar period of diagnosis in SEER and TCR respectively.

Most patients received surgery (SEER: 81.3%, TCR: 82.7%) and adjuvant radiation (SEER: 81.1%, TCR: 69.4%) as their first-line treatments. The proportion of each baseline characteristic except for sex was significantly different across the three calendar periods of diagnosis in SEER. For TCR, the distributions of age at diagnosis, race/ethnicity and radiation status differed significantly across P1, P2 and P3. Within each calendar period of diagnosis, the distributions of several factors varied significantly between SEER and TCR: P1, age at diagnosis (*P* < 0.001), race/ethnicity (*P* < 0.001), marital status (*P* = 0.021), tumor site (*P* = 0.002) and surgery (*P* = 0.001); P2, race/ethnicity (*P* < 0.001), surgery (*P* = 0.007) and radiation (*P* < 0.001); P3, race/ethnicity (*P* < 0.001), surgery (*P* = 0.021) and radiation (*P* < 0.001).

### Survival results in SEER, TCR and the combined dataset

As shown in Table 2, the median OS for P1, P2 and P3 were 8, 10 and 11 months in SEER (log-rank test, *P* < 0.001), 9, 10 and 11 months in TCR (log-rank test, *P* < 0.001), and 8, 10 and 11 months in the combined dataset (log-rank test, *P* < 0.001), respectively. The differences in OS of P1 versus P2 (log-rank test, *P* < 0.001), P1 versus P3 (log-rank test, *P* < 0.001), and P2 versus P3 (log-rank test, *P* < 0.001) were statistically significant in SEER and the combined dataset. Analysis of the TCR dataset revealed a significant improved survival from P1 to P2, and from P1 to P3, but no significant effect from P2 to P3 was detected (log-rank test, *P* = 0.833).

**Table 2:** Median OS, 1-year and 2-year survival rate by calendar period of diagnosis in SEER, TCR and the combined dataset*

	Calendar Period of Diagnosis							
Survival statistics	Jan 2000 - Feb 2005 (P1)	Mar 2005 - Apr 2009 (P2)	May 2009 - Dec 2013 (P3)	Total (P1, P2 and P3)	P1 vs P2	P1 vs P3	P2 vs P3	Trend Test
**SEER**					*P^a^*	*P^b^*	*P^c^*	*P^d^*
Total cases, N	8169	7420	8989	24578	<0.001	<0.001	<0.001	<0.001
Death cases, N (%)	7960 (97.4)	7045 (94.9)	6359 (70.7)	21364 (86.9)				
Median OS (months, IQR)	8.0 (3.0-16.0)	10.0 (4.0-20.0)	11.0 (4.0-21.0)	10.0 (4.0-19.0)				
1-year survival rate, % (95%CI)	33.6 (32.6-34.7)	42.1 (41.0-43.3)	45.1 (43.9-46.2)	40.0 (39.4-40.6)	<0.001	<0.001	<0.001	
2-year survival rate, % (95%CI)	12.6 (11.9-13.4)	18.8 (17.9-19.7)	19.8 (18.7-20.9)	16.6 (16.1-17.1)	<0.001	<0.001	0.070	
**TCR**								
Total cases, N	1357	1286	1712	4355	<0.001	<0.001	0.833	<0.001
Death cases, N (%)	1318 (97.1)	1219 (94.8)	1242 (72.5)	3779 (86.8)				
Median OS (months, IQR)	9.0 (4.0-16.0)	10.0 (4.0-20.9)	11.0 (4.0-20.0)	10.0 (4.0-19.0)				
1-year survival rate, % (95%CI)	36.1 (33.6-38.7)	42.5 (39.8-45.2)	44.5 (41.8-47.1)	41.0 (39.5-42.5)	<0.001	<0.001	0.306	
2-year survival rate, % (95%CI)	13.3 (11.6-15.2)	20.4 (18.3-22.7)	19.4 (17.0-21.9)	17.5 (16.3-18.8)	<0.001	<0.001	0.571	
**The combined dataset**								
Total cases, N	9526	8706	10701	28933	<0.001	<0.001	<0.001	<0.001
Death cases, N (%)	9278 (97.4)	8264 (94.9)	7601 (71.0)	25143 (86.9)				
Median OS (months, IQR)	8.0 (4.0-16.0)	10.0 (4.0-20.0)	11.0 (4.0-21.0)	10.0 (4.0-19.0)				
1-year survival rate, % (95%CI)	34.0 (33.0-34.9)	42.2 (41.2-43.2)	45.0 (43.9-46.0)	40.2 (39.6-40.7)	<0.001	<0.001	<0.001	
2-year survival rate, % (95%CI)	12.7 (12.1-13.4)	19.1 (18.2-19.9)	19.8 (18.8-20.8)	16.8 (16.3-17.2)	<0.001	<0.001	0.150	
**SEER vs TCR**								
Log-rank test, *P* value	0.265	0.874	0.021	0.657				

Abbreviation: OS, overall survival; IQR, interquartile range; 95%CI, 95% confidence interval.

*: Using log-rank test tested the differences in Kaplan–Meier survival functions across calendar periods of diagnosis.

a: *P* value for comparison of survival functions, and two-sided Chi-square test with Schouten correction in 1-year or 2-year survival rate between P1 and P2 in SEER or TCR or the combined dataset.

b: *P* value for comparison of survival functions, and two-sided Chi-square test with Schouten correction in 1-year or 2-year survival rate between P1 and P3 in SEER or TCR or the combined dataset.

c: *P* value for comparison of survival functions, and two-sided Chi-square test with Schouten correction in 1-year or 2-year survival rate between P2 and P3 in SEER or TCR or the combined dataset.

d: Trend *P* value for survival functions across P1, P2 and P3 in SEER or TCR or the combined dataset.

After performing Chi-square test with Schouten correction, 1-year survival rate increased significantly over the three calendar periods and reached a peak in P3 in SEER (P1: 33.6%, P2: 42.1%, and P3: 45.1%; *P* < 0.001 for P1 versus P2, P1 versus P3, and P2 versus P3) and the combined dataset (P1: 34.0%, P2: 42.2%, and P3: 45.0%; *P* < 0.001 for P1 versus P2, P1 versus P3, and P2 versus P3). For the TCR dataset, a significant improvement was found in 1-year survival rate from P1 to P2 (P1: 36.1%, P2: 42.5%; *P* < 0.001 for P1 versus P2), from P1 to P3 (P1: 36.1%, P3: 44.5%; log-rank test, *P* < 0.001 for P1 versus P3), but no significant survival rate difference was observed from P2 to P3 (P2: 42.5%, P3: 44.5%; *P* = 0.306 for P2 versus P3). Interestingly, from P1 to P2, 2-year survival rate elevated significantly in SEER (P1: 12.6%, P2: 18.8%), TCR (P1: 13.3%, P2: 20.4%) and the combined dataset (P1: 12.7%, P2: 19.1%), while no significant difference in 2-year survival rate between P2 and P3 was found in all three datasets (Table 2).

**Figure 1:**
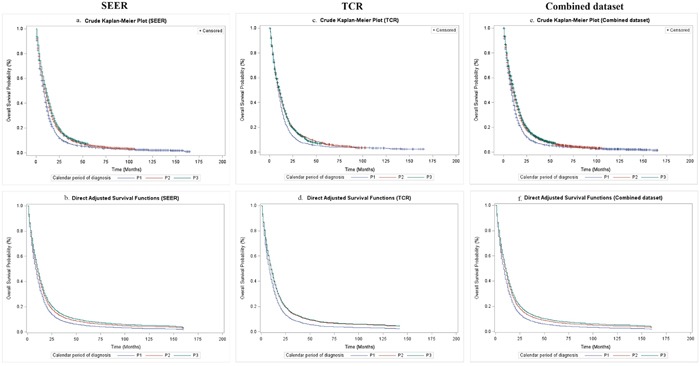
Overall survival of GBM patients by calendar period of diagnosis in SEER, TCR and the combined dataset **(a)** Crude overall survival in SEER; **(b)** Direct adjusted survival after adjusting covariates in SEER; **(c)** Crude overall survival in TCR; **(d)** Direct adjusted survival after adjusting covariates in TCR; **(e)** Crude overall survival in the combined dataset; **(f)** Direct adjusted survival after adjusting covariates in the combined dataset.

Comparing survival functions between SEER and TCR, the results from the two datasets revealed no significant difference in P1 (log-rank test, *P* = 0.265) and P2 (log-rank test, *P* = 0.874), but varied significantly in P3 (log-rank test, *P* = 0.021). The log-rank tests for a trend of OS over calendar period of diagnosis were significant in SEER, TCR and the combined dataset (Table 2 and Figure 1). Since the TCR dataset offers patients with longer follow-up period up to May 2015, we did a sub-analysis of the dataset including the extended follow-up period. It revealed a prolonged median OS in P3 (12.0 months) and significantly improved survival during P3 period (P2 versus P3, *P* < 0.001) (Supplementary Table 2 and Supplementary Figure 1).

Table 3, Table 4 and Table 5 present the hazard ratios (HRs) and 95% confidence intervals (95% CI) from Cox proportional hazards regression models in SEER, TCR and the combined dataset, respectively. The HRs for calendar period of diagnosis did not change after controlling for different sets of covariates in multivariate models (Model 1, Model 2 and Model 3 for SEER and TCR; Model 1, Model 2, Model 3 and Model 4 for the combined dataset), except for the TCR dataset. Based on the analysis of the TCR database, the risk for death during P3 was higher than that of P2 in age-sex adjusted model, Model 1 and Model 2, but it decreased to 0.78 (HR: 0.78, 95% CI: 0.72-0.84) when adding surgery and radiation as shown in Model 3 (Table 4). With P1 serving as a reference group as shown in Model 3, there was a significant reduction of risks of death during P2 and P3 periods in SEER (P2, HR: 0.83, 95% CI: 0.81–0.86; P3, HR: 0.77, 95% CI: 0.74-0.79) and the combined dataset (P2, HR: 0.82, 95% CI: 0.80–0.85; P3, HR: 0.76, 95% CI: 0.73-0.78). For the combined dataset, we built up Model 4 by adding “cancer registries” in the covariates of Model 3, and found that the HRs during P2 and P3 were unchanged comparing results in Model 3. Additionally, the risks of death derived from age-sex adjusted model, Model 1, Model 2 and Model 3 remained stable in TCR with extended follow-up (Supplementary Table 3).

**Table 3:** Risk of death among GBM patients in relation to calendar period of diagnosis in SEER (N=24578)

	Cases	Death	Age-sex adjusted		Model 1^a^		Model 2^b^		Model 3^c^	
Predictors	N	N	HR	95%CI	HR	95%CI	HR	95%CI	HR	95%CI
Calendar period of diagnosis										
Jan 2000-Feb 2005 (P1)	8169	7960	1.00	-	1.00	-	1.00	-	1.00	-
Mar 2005-Apr 2009 (P2)	7420	7045	0.82	0.79 - 0.85	0.82	0.79 - 0.85	0.83	0.80 - 0.85	0.83	0.81 - 0.86
May 2009-Dec 2013 (P3)	8989	6359	0.74	0.72 - 0.76	0.74	0.72 - 0.77	0.75	0.72 - 0.77	0.77	0.74 - 0.79
Age at diagnosis group										
20-49	4379	3401	1.00	-	1.00	-	1.00	-	1.00	-
50-59	6284	5321	1.50	1.44 - 1.57	1.51	1.44 - 1.58	1.50	1.44 - 1.57	1.50	1.44 - 1.57
60-69	7040	6186	2.02	1.94 - 2.11	2.03	1.94 - 2.12	2.03	1.95 - 2.12	2.00	1.92 - 2.09
70-	6875	6456	3.41	3.27 - 3.56	3.39	3.24 - 3.54	3.39	3.24 - 3.54	3.12	2.98 - 3.26
Sex										
Male	14432	12531	1.00	-	1.00	-	1.00	-	1.00	-
Female	10146	8833	0.97	0.94 - 0.99	0.94	0.91 - 0.96	0.94	0.91 - 0.96	0.94	0.92 - 0.97
Race/Ethnicity										
White	19768	17399			1.00	-	1.00	-	1.00	-
Black	1341	1134			1.06	1.00 - 1.13	1.06	0.99 - 1.12	1.02	0.96 - 1.08
Hispanic	2344	1938			0.98	0.93 - 1.02	0.97	0.92 - 1.02	0.94	0.89 - 0.98
Others	1125	893			0.83	0.78 - 0.89	0.82	0.77 - 0.88	0.80	0.75 - 0.86
Marital status										
Single	3259	2667			1.00	-	1.00	-	1.00	-
Married	16671	14468			0.90	0.86 - 0.93	0.89	0.86 - 0.93	0.95	0.91 - 0.99
DWS	4648	4229			1.09	1.04 - 1.15	1.09	1.04 - 1.15	1.11	1.06 - 1.17
Tumor site										
Supratentorial	19005	16380					1.00	-	1.00	-
Infratentorial/NOS	5573	4984					1.24	1.20 - 1.28	1.15	1.11 - 1.18
Surgery										
No surgery	4596	4332							1.00	-
Local excision/biopsy	4872	4295							0.61	0.58 - 0.63
Partial resection	7073	6111							0.65	0.63 - 0.68
GTR	8037	6626							0.49	0.47 - 0.51
Radiation										
Untreated	4649	4386							1.00	-
Treated	19929	16978							0.51	0.49 - 0.52

Abbreviation: DWS, divorced or widowed or separated; NOS, not otherwise specified; GTR, gross total resection; HR, hazard ratio; 95%CI, 95% confidence interval.

a: Model 1: Adjusted age at diagnosis, sex, race/ethnicity and marital status.

b: Model 2: Adjusted age at diagnosis, sex, race/ethnicity, marital status and tumor site.

c: Model 3: Adjusted age at diagnosis, sex, race/ethnicity, marital status, tumor site, surgery and radiation.

**Table 4:** Risk of death among GBM patients in relation to calendar period of diagnosis in TCR (N=4355)

	Cases	Death	Age-sex adjusted		Model 1^a^		Model 2^b^		Model 3^c^	
Predictors	N	N	HR	95%CI	HR	95%CI	HR	95%CI	HR	95%CI
Calendar period of diagnosis										
Jan 2000-Feb 2005 (P1)	1357	1318	1.00	-	1.00	-	1.00	-	1.00	-
Mar 2005-Apr 2009 (P2)	1286	1219	0.81	0.75 - 0.88	0.81	0.75 - 0.88	0.81	0.75 - 0.88	0.79	0.73 - 0.86
May 2009-Dec 2013 (P3)	1712	1242	0.83	0.76 - 0.89	0.83	0.77 - 0.90	0.83	0.77 - 0.90	0.78	0.72 - 0.84
Age at diagnosis group										
20-49	801	621	1.00	-	1.00	-	1.00	-	1.00	-
50-59	1173	996	1.46	1.32 - 1.62	1.45	1.31 - 1.60	1.44	1.30 - 1.60	1.41	1.28 - 1.56
60-69	1270	1115	1.93	1.75 - 2.13	1.91	1.73 - 2.11	1.91	1.72 - 2.11	1.86	1.68 - 2.06
70-	1111	1047	3.18	2.88 - 3.53	3.12	2.81 - 3.46	3.13	2.82 - 3.48	2.88	2.59 - 3.20
Sex										
Male	2607	2257	1.00	-	1.00	-	1.00	-	1.00	-
Female	1748	1522	1.02	0.95 - 1.08	1.00	0.93 - 1.07	1.00	0.94 - 1.07	0.99	0.92 - 1.05
Race/Ethnicity										
White	3274	2902			1.00	-	1.00	-	1.00	-
Black	269	232			1.05	0.92 - 1.20	1.04	0.91 - 1.19	1	0.87 - 1.14
Hispanic	715	577			0.91	0.83 - 1.00	0.89	0.82 - 0.98	0.86	0.79 - 0.94
Others	97	68			0.74	0.58 - 0.94	0.73	0.57 - 0.93	0.68	0.54 - 0.87
Marital status										
Single	609	494			1.00	-	1.00	-	1.00	-
Married	3022	2639			0.97	0.88 - 1.07	0.98	0.89 - 1.08	1.03	0.94 - 1.14
DWS	724	646			1.10	0.98 - 1.25	1.12	0.99 - 1.26	1.16	1.03 - 1.31
Tumor site										
Supratentorial	3423	2961					1.00	-	1.00	-
Infratentorial/NOS	932	818					1.25	1.16 - 1.35	1.15	1.06 - 1.24
Surgery										
No surgery	753	686							1.00	-
Local excision/biopsy	877	782							0.65	0.59 - 0.73
Partial resection	1164	1005							0.73	0.66 - 0.80
GTR	1561	1306							0.56	0.51 - 0.61
Radiation										
Untreated	1332	1157							1.00	-
Treated	3023	2622							0.71	0.66 - 0.77

Abbreviation: DWS, divorced or widowed or separated; NOS, not otherwise specified; GTR, gross total resection; HR, hazard ratio; 95%CI, 95% confidence interval.

a: Model 1: Adjusted age at diagnosis, sex, race/ethnicity and marital status.

b: Model 2: Adjusted age at diagnosis, sex, race/ethnicity, marital status and tumor site.

c: Model 3: Adjusted age at diagnosis, sex, race/ethnicity, marital status, tumor site, surgery and radiation.

**Table 5:** Risky of death among GBM patients in relation to calendar period of diagnosis in the combined dataset (N=28933)

	Cases	Death	Age-sex adjusted		Model 1^a^		Model 2^b^		Model 3^c^		Model 4^d^	
Predictors	N	N	HR	95%CI	HR	95%CI	HR	95%CI	HR	95%CI	HR	95%CI
Calendar period of diagnosis												
Jan 2000-Feb 2005 (P1)	9526	9278	1.00	-	1.00	-	1.00	-	1.00	-	1.00	-
Mar 2005-Apr 2009 (P2)	8706	8264	0.82	0.79 - 0.84	0.82	0.79 - 0.84	0.82	0.80 - 0.85	0.82	0.80 - 0.85	0.82	0.80 - 0.85
May 2009-Dec 2013 (P3)	10701	7601	0.75	0.73 - 0.77	0.75	0.73 - 0.78	0.76	0.73 - 0.78	0.76	0.73 - 0.78	0.76	0.73 - 0.78
Age at diagnosis group												
20-49	5180	4022	1.00	-	1.00	-						
50-59	7457	6317	1.50	1.44 - 1.56	1.50	1.44 - 1.56	1.49	1.44 - 1.56	1.48	1.43 - 1.55	1.49	1.43 - 1.55
60-69	8310	7301	2.01	1.93 - 2.09	2.01	1.94 - 2.10	2.02	1.94 - 2.10	1.98	1.90 - 2.06	1.98	1.90 - 2.06
70-	7986	7503	3.40	3.26 - 3.53	3.36	3.22 - 3.50	3.36	3.23 - 3.50	3.09	2.96 - 3.22	3.09	2.96 - 3.22
Sex												
Male	17039	14788	1.00	-	1.00	-						
Female	11894	10355	0.97	0.95 - 1.00	0.95	0.92 - 0.97	0.95	0.92 - 0.97	0.95	0.93 - 0.97	0.95	0.93 - 0.97
Race/Ethnicity												
White	23042	20301			1.00	-						
Black	1610	1366			1.06	1.01 - 1.12	1.06	1.00 - 1.12	1.02	0.96 - 1.07	1.02	0.96 - 1.08
Hispanic	3059	2515			0.96	0.92 - 1.00	0.95	0.92 - 0.99	0.91	0.87 - 0.95	0.92	0.88 - 0.96
Others	1222	961			0.82	0.77 - 0.88	0.81	0.76 - 0.87	0.79	0.74 - 0.84	0.79	0.74 - 0.84
Marital status												
Single	3868	3161										
Married	19693	17107			0.91	0.87 - 0.94	0.91	0.87 - 0.94	0.96	0.92 - 1.00	0.96	0.92 - 1.00
DWS	5372	4875			1.10	1.05 - 1.15	1.10	1.05 - 1.15	1.13	1.08 - 1.18	1.13	1.08 - 1.18
Tumor site												
Supratentorial	22428	19341										
Infratentorial/NOS	6505	5802					1.24	1.21 - 1.28	1.14	1.11 - 1.18	1.14	1.11 - 1.18
Surgery												
No surgery	5349	5018										
Local excision/biopsy	5749	5077							0.61	0.59 - 0.64	0.61	0.59 - 0.64
Partial resection	8237	7116							0.66	0.64 - 0.69	0.66	0.64 - 0.69
Gross subtotal resection (GTR)	9598	7932							0.50	0.48 - 0.52	0.50	0.48 - 0.52
Radiation												
Untreated	5981	5543										
Treated	22952	19600							0.55	0.53 - 0.56	0.54	0.52 - 0.56
Cancer Registries												
TCR	4355	3779										
SEER	24578	21364									1.07	1.03 - 1.11

Abbreviation: DWS, divorced or widowed or separated; NOS, not otherwise specified; GTR, gross total resection; HR, hazard ratio; 95%CI, 95% confidence interval.

a: Model 1: Adjusted age at diagnosis, sex, race/ethnicity and marital status.

b: Model 2: Adjusted age at diagnosis, sex, race/ethnicity, marital status and tumor site.

c: Model 3: Adjusted age at diagnosis, sex, race/ethnicity, marital status, tumor site, surgery and radiation.

d: Model 4: Adjusted age at diagnosis, sex, race/ethnicity, marital status, tumor site, surgery, radiation and cancer registries.

**Figure 2:**
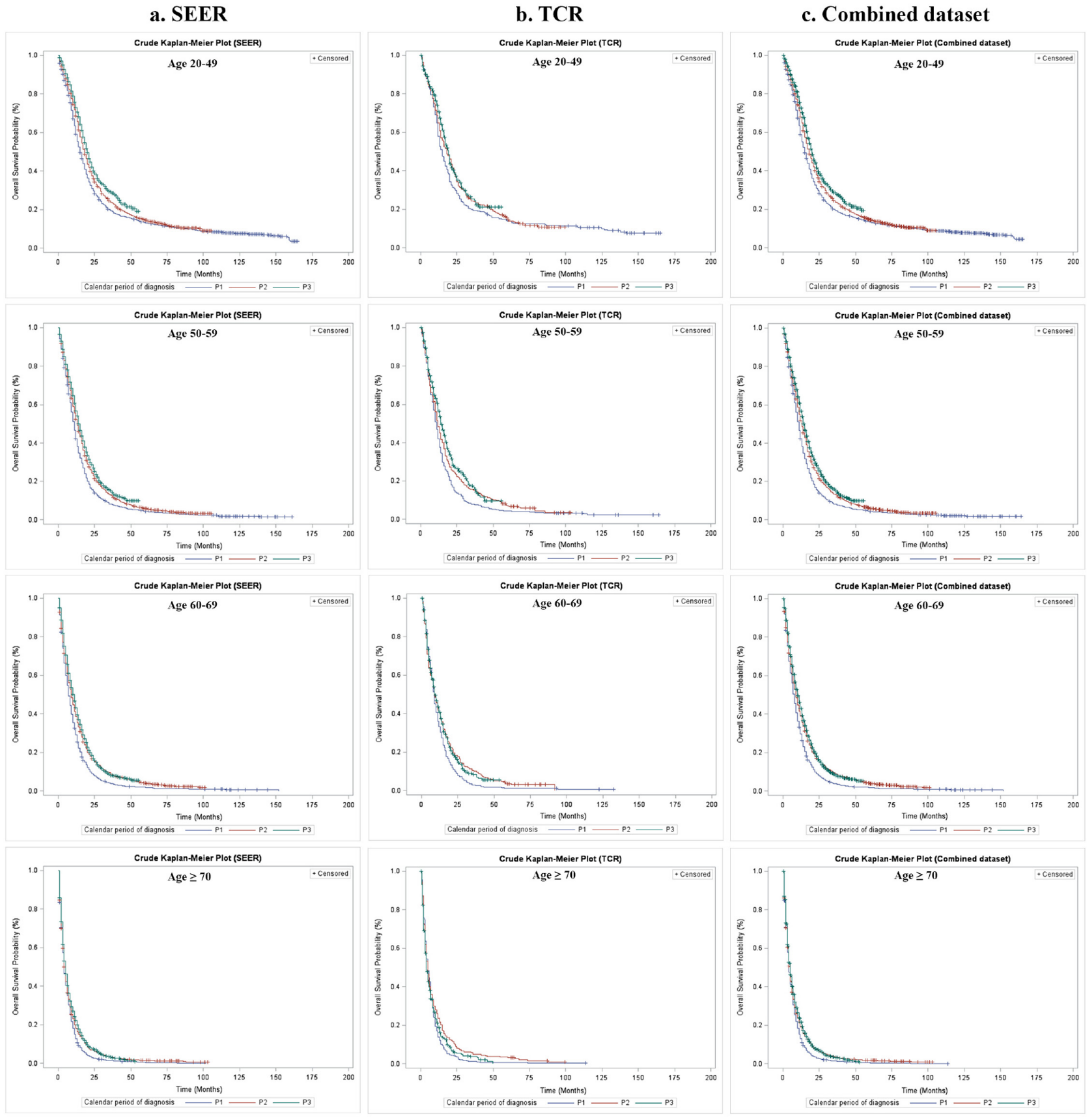
Overall survival of GBM patients by calendar period of diagnosis and age group at diagnosis in SEER, TCR and the combined dataset, Kaplan–Meier survival estimates **(a)** Crude overall survival stratified by age group at diagnosis in SEER; **(b)** Crude overall survival stratified by age group at diagnosis in TCR; **(c)** Crude overall survival stratified by age group at diagnosis in the combined dataset.

**Figure 3:**
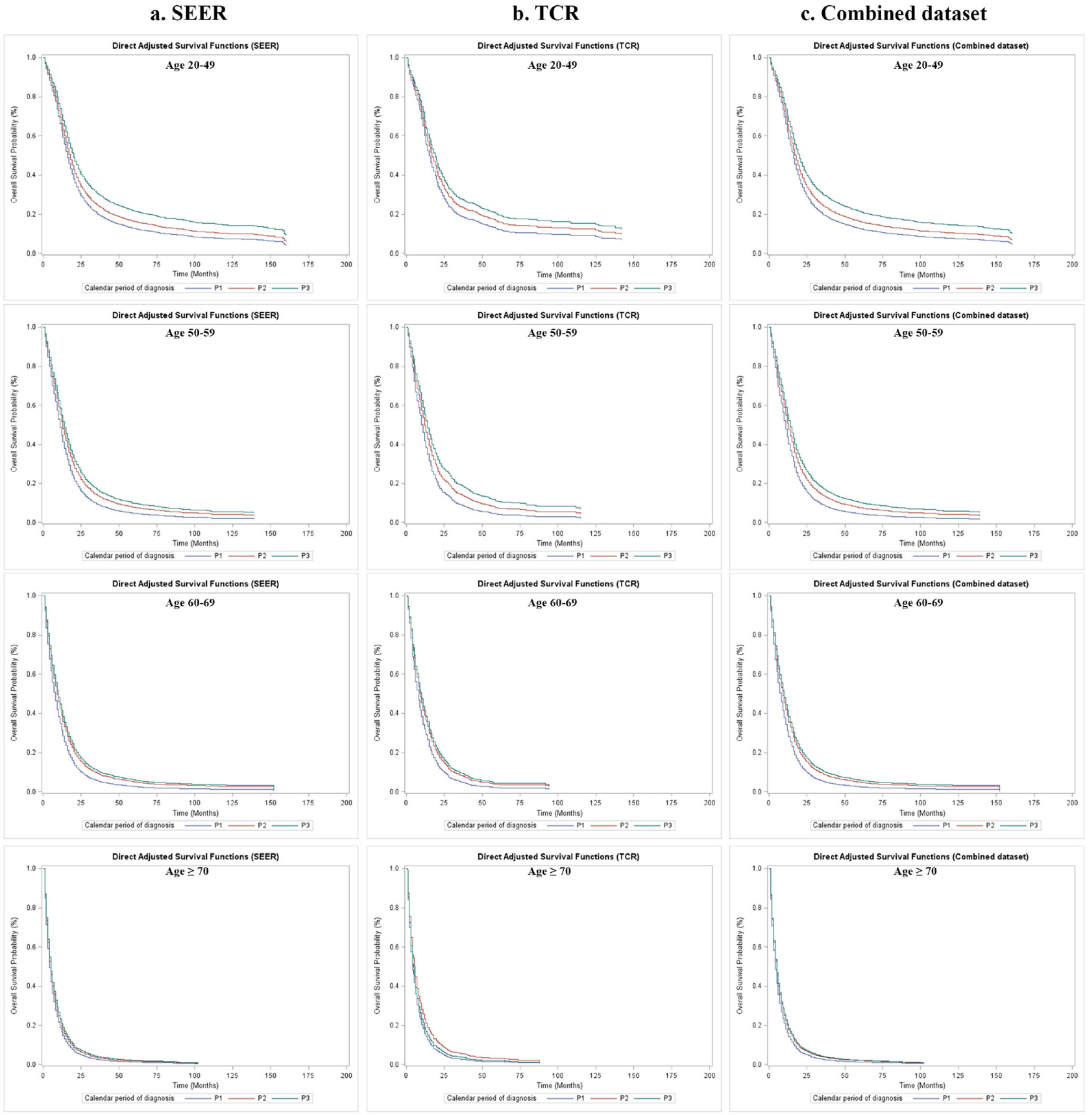
Overall survival of GBM patients by calendar period of diagnosis and age group at diagnosis in SEER, TCR and the combined dataset, direct adjusted survival functions **(a)** Direct adjusted survival after adjusting covariates stratified by age group at diagnosis in SEER; **(b)** Direct adjusted survival after adjusting covariates stratified by age group at diagnosis in TCR; **(c)** Direct adjusted survival after adjusting covariates stratified by age group at diagnosis in the combined dataset.

The crude Kaplan–Meier survival estimates and direct adjusted survival results based on Model 3 (SEER and TCR) or Model 4 (the combined dataset) are presented in Figure 1. Figure 2 and Figure 3 displayed survival curves stratified by age group at diagnosis. The trend and pattern of survival were consistent across three datasets, but the survival curve for P3 was nearly identical to that of P2 in TCR (Figure 1). After stratification by age group, survival distributions became worse with advancing age, but the improved survival across calendar periods was still preserved within each stratum of age groups in SEER and the combined dataset (Figure 2 and Figure 3). However, the survival curve during P2 was superior to that of P3 among the group of aged ≥ 70 in TCR. Similar patterns of survival were observed in TCR with extended follow-up, except the survival curve during P3 which was identical to that of P2 in the elderly patients (aged ≥ 70) (Supplementary Figure 2). In stratification analyses by age at diagnosis, tumor site, surgery and radiation status, the decreased HR over calendar period within each stratum of stratified variables was observed in SEER, TCR, the combined dataset (Table 6) and TCR with extended follow-up (Supplementary Table 4). In addition, we have conducted a sub-analysis in an analytic cohort (aged ≥ 18 years old), and the results were consistent with the findings presented above (Data not shown).

**Table 6:** Risk of death in GBM patients by calendar period of diagnosis and by time-varying covariates*

			SEER						TCR						The combined dataset			
	P1		P2		P3		P1		P2		P3		P1		P2		P3	
Time-varying covariates	HR	95%CI	HR	95%CI	HR	95%CI	HR	95%CI	HR	95%CI	HR	95%CI	HR	95%CI	HR	95%CI	HR	95%CI
Age group at diagnosis																		
20-49	1.00	-	0.87	0.81 - 0.94	0.73	0.67 - 0.80	1.00	-	0.86	0.71 - 1.04	0.76	0.61 - 0.94	1.00	-	0.87	0.81 - 0.94	0.73	0.68 - 0.80
50-59	1.00	-	0.82	0.77 - 0.87	0.73	0.68 - 0.78	1.00	-	0.80	0.69 - 0.93	0.68	0.58 - 0.79	1.00	-	0.81	0.76 - 0.85	0.71	0.66 - 0.75
60-69	1.00	-	0.80	0.75 - 0.85	0.74	0.70 - 0.79	1.00	-	0.82	0.70 - 0.95	0.77	0.66 - 0.89	1.00	-	0.80	0.75 - 0.84	0.74	0.70 - 0.78
70-	1.00	-	0.89	0.84 - 0.95	0.85	0.80 - 0.90	1.00	-	0.78	0.67 - 0.91	0.91	0.78 - 1.06	1.00	-	0.87	0.82 - 0.92	0.85	0.80 - 0.89
Tumor site																		
Supratentorial	1.00	-	0.83	0.80 - 0.86	0.77	0.74 - 0.80	1.00	-	0.76	0.70 - 0.83	0.75	0.68 - 0.82	1.00	-	0.81	0.79 - 0.84	0.75	0.73 - 0.78
Infratentorial/NOS	1.00	-	0.84	0.78 - 0.90	0.76	0.71 - 0.81	1.00	-	0.78	0.31 - 1.95	0.74	0.30 - 1.81	1.00	-	0.78	0.57 - 1.06	0.76	0.55 - 1.04
Surgery																		
No surgery	1.00	-	0.89	0.83 - 0.95	0.84	0.78 - 0.91	1.00	-	0.90	0.74 - 1.08	0.95	0.79 - 1.15	1.00	-	0.89	0.84 - 0.95	0.83	0.77 - 0.88
Local excision/biopsy	1.00	-	0.90	0.83 - 0.97	0.92	0.86 - 0.99	1.00	-	0.77	0.64 - 0.92	0.72	0.60 - 0.86	1.00	-	0.85	0.79 - 0.91	0.83	0.78 - 0.89
Partial resection	1.00	-	0.79	0.74 - 0.84	0.74	0.69 - 0.78	1.00	-	0.80	0.68 - 0.93	0.70	0.60 - 0.82	1.00	-	0.77	0.73 - 0.82	0.70	0.66 - 0.74
GTR	1.00	-	0.89	0.84 - 0.94	0.80	0.75 - 0.85	1.00	-	0.77	0.68 - 0.88	0.78	0.68 - 0.89	1.00	-	0.82	0.78 - 0.86	0.73	0.69 - 0.77
Radiation																		
Untreated	1.00	-	0.97	0.90 - 1.04	0.93	0.86 - 1.00	1.00	-	0.85	0.72 - 1.00	0.76	0.65 - 0.88	1.00	-	0.96	0.90 - 1.02	0.90	0.84 - 0.96
Treated	1.00	-	0.79	0.76 - 0.82	0.72	0.69 - 0.75	1.00	-	0.78	0.71 - 0.85	0.81	0.73 - 0.89	1.00	-	0.79	0.77 - 0.82	0.73	0.71 - 0.76

Abbreviation: Calendar period of diagnosis, including P1, P2 and P3 (P1, Jan 2000-Feb 2005; P2, Mar 2005-Apr 2009; P3, May 2009-Dec 2013); NOS, not otherwise specified; GTR, gross total resection; HR, hazard ratio; 95%CI, 95% confidence interval.

*: Adjusted age at diagnosis, sex, race/ethnicity, marital status, tumor site, surgery, radiation and cancer registries as appropriate.

## DISCUSSION

Prior analysis of RCTs [11], [30] and population-based studies demonstrated that administration of TMZ significantly improved the OS for patients with GBM [21], [22], [23], [24], [25], [31]. Since FDA accelerated approval of BEV for progressive GBM on May 9, 2009, two SEER-based studies focused on the impact of BEV on OS in general GBM population [28], [29]. Both studies suggested that the addition of BEV to GBM treatment improved OS among GBM patients. However, the two studies had limited methods of categorizing patients and statistical analyses, limited number of GBM patients and short follow-up period after FDA approval of BEV. Therefore, more new investigations of general GBM patients in “real-world” setting with longer period of follow-up are needed to examine the effect of BEV on OS of GBM patients after FDA approval.

Our findings suggested that OS and 1-year survival rate improved significantly across calendar period of diagnosis in SEER, TCR and the combined dataset, except that the improved survival within P3 compared to P2 in TCR was not significant (*P* = 0.833). There was no survival benefit observed in 1-year or 2-year survival rate between P2 and P3 in TCR, nor 2-year survival rate between P2 and P3 in SEER and in the combined dataset. One explanation for the reduced survival in P3 comparing to P2 in TCR could be related to the lower proportion of patients received radiation during P3. The proportion of patients treated with radiation therapy in TCR decreased from 80.8% to 69.8% and to 60.1% in P1, P2, and P3, successively. However, when we did a sub-analysis of the extended follow-up of the TCR dataset up to May 2015, we found that the difference in survival between P2 and P3 was significant (Supplementary Figure 1 and Supplementary Table 2). This finding indicated that the survival benefit of BEV could be observed when given longer period of follow-up and that may compensate the loss of benefit with lower radiation proportion in P3. Another factor may contribute to the improved GBM survival observed in P3 with extended follow-up period was the availability of broader insurance coverage in Texas, which may translate to more applications of BEV in 2014 and 2015. Since the implementation of Affordable Care Act (ACA) Marketplace in Texas in January 2014, the uninsured rate in Texas dropped from 22.1% (2013) to 19.1% (2014), while the uninsured rate in Texas remained the highest across the nation. The uninsured rate nationally reduced from 14.5% (2013) to 11.7% (2014) [32], [33].

While analyzing the cohorts according to different age groups at diagnosis, we found that the survival benefit across P1, P2 and P3 decreased with increasing age: the patients at 20-49 years old group had the most improved survival in P3 comparing to P2, but the elderly GBM patients (age at diagnosis ≥ 70 years old) showed limited OS benefit in SEER and even reduced survival in TCR from P2 to P3 periods. This diminished OS benefit along with advancing age is intriguing. It could be related to three possible explanations: first, for elderly patients (age at diagnosis ≥ 70 years), physicians might not prescribe SOC due to relatively shorter life expectancy considering their ages and limited tolerance, and may apply to standard or short course radiation only with avoiding using TMZ or BEV or both. Second, *IDH1/2* mutations occur more frequently in younger patients, and these patients have an improved OS than those with wild-type *IDH* genes which is the case for nearly all *de novo* GBM of the elderly [34], [35]. However, these two reasons are less likely to be valid since there is no known evidence of changes of physician treatment routine for GBM patients except FDA approval of new therapies, nor gene pool alteration of GBM patients over the past 13 years. The third reason is the most likely explanation of this observation in our opinion. It is related to different mechanisms of GBM tumor pathogenesis and responses to BEV between young and elderly patients. By performing retrospective analysis of the AVAglio trial, Sandmann et al revealed that GBM patients with *IDH1* mutation and PN subtype were more likely to obtain OS benefit from BEV therapy during first-line treatment [36]. Given the fact that elderly GBM patients are more likely to be *IDH1* wild-type and Mes subtype of GBM than younger patients [34], [35], [36], they may not draw survival benefit with BEV therapy as younger patients do (see P3, Figure 2, Figure 3 and Table 6). The disparity of deriving survival benefit from BEV in different age groups reminds us that personalized strategy of GBM treatment, such as drug selection, is critical for improving GBM patient's outcomes in the future.

The survival patterns between SEER and TCR were similar across calendar period of diagnosis in Figure 1, but differed significantly within P3 by applying the log-rank test. This difference might be attributed to the smaller sample size, reduced proportion of radiation received in TCR population as mentioned above or other potential factors we cannot obtain detailed data in this study, including insurance coverage rate, socioeconomic status and access to health care resources in Texas versus the 18 regions of SEER registries [33]. Possible biological mechanisms for the impact of TMZ on the prolonging survival of GBM patients could be explained by the drug-related alkylation of DNA which interferes with GBM cell DNA replication, the depletion of *MGMT* repair enzyme activity [37], [38] or methylation of the *MGMT* promoter [5] after receiving the RT-TMZ. Two studies [39], [40] indicated that BEV has glucocorticoid-like and steroid-sparing effects which was nicknamed as “super-steroid” and has resulted in diminished glucocorticoids dosage or need, reduction in edema or possibly in tumor size. However, it is unlikely that steroid-like effect alone can produce such significant durable survival benefit observed in all three cohorts for the follow-up duration of 4 years since FDA approval in 2009. We believe BEV alone or in combination with TMZ or other chemo-radiation therapies have a therapeutic effect on extending survival of GBM patients.

Median OS and 1-year or 2-year survival rate in our study were lower than those reported from previous clinical trials in GBM patients including RT-TMZ [11], Dose-Dense (DD) TMZ [41], TMZ+TTFields (Novo-TTF device) [42], BEV [27], BEV+RT-TMZ [26] or Rindopepimut/GM-CSF (no results available) (Supplementary Table 1). Considering the TMZ effect on GBM survival, the median OS was 10 months within P2 (post-RT-TMZ and pre-BEV) for all three datasets (SEER, TCR and the combined dataset). This was a 30-40% reduction comparing the median OSs reported in the EORTC-NCIC trial (EORTC 26981/22981: NCIC CE.3 intergroup trial, 14.6 months with RT-TMZ) [11], RTOG 0525 trial (16.6 months in control arm with SOC) [41] and EF-14 trial (15.6 months in control arm with SOC) [42]. Two-year survival rate during P2 (SEER: 18.8%, TCR: 20.4% and the combined dataset: 19.1%) was lower than those reported in RCTs (26.5% in EORTC-NCIC trial [11], 34.2% in RTOG 0525 trial [41] and 29% in EF-14 trial [42]) (Supplementary Table 1).

A similar pattern was observed for the impact of BEV on GBM survival given our result of median OS during P3 (11.0 months) was approximate 30-35% lower than that of RTOG 0825 trial (15.7 months in BEV arm)[27] and AVAglio (BO21990) trial (16.8 months with BEV+RT-TMZ) [26]. In the present study, 1-year survival rate was improved significantly from P2 to P3 in SEER and the combined dataset, but significant improvement in 2-year survival rate from P2 to P3 was not detected. These findings were in accord with Chinot et al study [26] (1-year survival rate was significantly improved in BEV+RT-TMZ arm, *P* = 0.049, but no significance found in 2-year survival rate between BEV+RT-TMZ arm and Placebo+RT-TMZ arm, *P* = 0.240). The apparent difference in survival between our study and prior RCTs might be explained by selection bias favoring RCTs. For example, all clinical trials patients were selected with good performance status, younger age ranges, adequate hematologic, cardiovascular, renal, and hepatic function without significant comorbidity [11], [26], [27], [41], [42] (Supplementary Table 1).

By contrast, the results in our study were comparable to the median OS and survival rate presented in the population-based studies by using SEER, VHA and NPD (Norwegian Prescription Database) linked CRN (Cancer Registry of Norway), even though most studies applied different classification of time periods [21], [23], [24], [25], [28], [29], [31]. The majority of studies on TMZ set 2005 as the cut-off between pre-TMZ and post-TMZ eras based on SEER [21], [24] and their results were consistent with our findings within P1 and P2 periods (Supplementary Table 1). Two other studies were based on the VHA database in the U.S. and NPD linked CRN in Norway, respectively, which were independent cohorts from SEER. These studies confirmed and substantiated the beneficial effect of TMZ on GBM survival after the introduction of TMZ concurrent with radiation and adjuvant TMZ. They reported similar median OS and survival rate to our findings.

Two other studies examined the potential survival benefit after the administration of BEV for GBM treatment in “real-world” settings. The conclusion in our study was in agreement with the findings reported by Johnson et al [28]. But they ascertained the time window based on three years of death records and demonstrated that the median OS for the patients deceased in 2006, 2008 and 2009 was 8, 7 and 9 months, respectively, whereas no 1-year or 2-year survival rates were presented and all analyses were performed by Mann-Whitney U tests or a log-rank test. Wachtel et al [29] reported 1-year survival rate of the time periods as 31.8% (Jan 2000-Jun 2003), 37.3% (Jul 2003-Mar 2005), 41.0% (Apr 2005–Oct 2007) and 43.0% (Nov 2007–Dec 2009), which was in consonance with the survival rates reported in our study. However, Wachtel et al defined BEV-TMZ era as “November 2007–December 2009” based on Vredenburgh et al study, a phase II trial result for BEV, which was published in October 2007 [43]. Further, they had no access to the sufficient study subjects who were diagnosed GBM after FDA approval of BEV since their time period of GBM diagnosis was Jan 2000 - Dec 2009. Therefore, they could not provide adequate evidence to prove the prolonged survival benefit after BEV FDA approval (Supplementary Table 1).

The strengths of the present study include: First, we addressed the research question by using two independent population-based datasets with larger sample sizes, longer follow-up periods comparing to previously reported studies [28], [29] and applicable adjustment of demographical and clinical covariates. Second, our paper is the first to explore the association between calendar period of GBM diagnosis and OS in TCR, which is an independent collection of GBM patients from SEER but using a similar standard method of data collection and management to SEER. Third, we also compared survival functions between SEER and TCR within each grouped calendar period and across all periods (including multi-comparison among P1, P2 and P3), and re-ran all the analyses based on the combined dataset and TCR with extended follow-up, which served as validation analyses and confirmed our initial observations. Fourth, in Dubrow et al study, the proportion of TMZ usage in the adjuvant chemotherapy increased from 28% (2001-2004) to 71% (2005-2008), and the median time to initiate TMZ therapy reduced to 1.0 months in 2005-2008 (lowest across all three time periods), which indicated that there is an speedy process of administration of TMZ after FDA approval and patients could obtain TMZ with minimal delay. Arvold et al [44] defined the TMZ era as June 1, 2005 - December 31, 2009, in SEER-Medicare database because they believed TMZ could be widely available in the US after FDA approval. In this regard, we believe FDA approval date can represent the start date of insurance coverage and physician's prescriptions with minimal delays. This is an appropriate proxy to the time-point that majority of qualified GBM patients began to receive TMZ and BEV, respectively. Although there is a delay during implementation of insurance coverage, the interval of delay is very short in this population since there are very limited options of therapies for GBM and the prognosis is extremely poor. This method of categorization could avoid uncertain transition periods and facilitate interpretation. Fifth, we performed stratification analysis, Cox proportional hazards model and direct adjusted survival curves by taking the potential time-varying covariates into account.

There are limitations of the current study: First, the present study was based on a retrospective cohort study, which means the difference in OS among the calendar period of diagnosis could be influenced by confounding factors. To limit the effects from confounders, we have adjusted varied combinations of covariates during the model selection and still achieved consistent results. Second, both SEER and TCR do not offer detailed clinical data, such as performance status, chemotherapy drugs received, numbers of craniotomies with or without Gliadel wafers carried out, salvage radiation or stereotactic radiosurgeries performed, molecular profiles (*MGMT* [41], *IDH1/2*) results, as well as direct causes of death, which may influence our ascertainment of OS. Third, other factors may contribute OS variations that are not available in SEER or TCR, including socioeconomic status, access to health insurance, complications or adverse events related to TMZ or BEV. The prior RCTs indicated that the usage of BEV would be associated with an elevated risk of side effects, such as hypertension, thromboembolic events, intestinal perforation or intracranial hemorrhage [26], [27]. Therefore, further studies including the above-mentioned aspects would be necessary to confirm the beneficial effect of TMZ and BEV on OS among GBM patients.

## CONCLUSIONS

Based on a population-based, retrospective study of two independent datasets, SEER and TCR as well as the pooled database from both datasets, we demonstrated that there was a significantly improved OS across the calendar period of GBM diagnosis from January 2000 to December 2013. In multivariate models, the survival benefit over calendar period remained unchanged after stratification. Although we cannot provide a direct causal relationship between concurrent TMZ with radiation, adjuvant TMZ for newly diagnosed GBM followed by BEV for recurrent GBM and successive increased survival in the thirteen years period based on a retrospective population-based analysis, the improved OS was likely resulted from the administrations of TMZ with radiation and adjuvant TMZ and then BEV after FDA approval, respectively.

## MATERIALS AND METHODS

### Study settings and populations

The latest database of the SEER Program (released on April 15, 2016) included cancer incidence and survival data across 18 population-based cancer registries in the United States (Connecticut, New Jersey, Detroit, Iowa, Kentucky, Louisiana, Atlanta, rural Georgia, greater Georgia, Hawaii, New Mexico, Seattle-Puget Sound, Utah, San Francisco-Oakland, San Jose-Monterey, Los Angeles, greater California, Alaska), covering approximately 30% of the U.S. population from January 2000 to December 2013. We requested access to the data and obtained permission from the National Cancer Institute (NCI), Division of Cancer Control and Population Sciences, Surveillance Research Program. The details of description, recruitment methodology, quality of control and follow-up protocols about SEER program were described elsewhere [45]. The TCR Limited-Use database for cancer diagnosed from 1995 to 2013, which is not included in the SEER database, has been developed since 1979 by the Texas Department of State Health Services, including demographics, clinical characteristics, and survival information. The variable settings of core data in TCR are similar to those collected in the SEER database and meet the high-quality data standards by National Program of Central Cancer Registries (NPCR), and Centers for Disease Control and Prevention [46].

Glioblastoma Multiforme (GBM) was defined by International Classification of Disease for Oncology, third edition (ICD-O-3) coded as 9440, 9441, or 9442 [21], [22], [24], [47], with topography codes C710-C719 and malignant brain neoplasm (behavior code was 3). Subjects were excluded due to the following criteria: age at diagnosis less than 20 years old; not primary tumors; patients diagnosed only from autopsy or from death certificate; not microscopically-confirmed; unknown race, marital status; the extent of surgery; or radiation therapy status and lack of survival data [21], [22], [23], [48], [49]. The final analytic dataset was limited to 24,578 and 4,355 GBM patients in SEER and TCR, respectively, and we also combined these two datasets for validation analysis. Since TCR offers longer follow-up period until May 2015, we did sub-analysis of P3 including the extended follow-up period and the results are reported in supplemental tables.

### Predictors, covariates, and outcome

Since detailed chemotherapy regimens were not available in SEER and TCR, we separated the study cohorts into three groups based on the FDA approval dates of TMZ as concurrent use with RT and then BEV, respectively: January 2000–February 2005 (pre-RT-TMZ and pre-BEV, P1), March 2005–April 2009 (post-RT-TMZ and pre-BEV, P2), and May 2009–December 2013 (post-RT-TMZ and post-BEV, P3). Covariates included age at diagnosis (20–49, 50–59, 60–69, ≥ 70 years), sex, race/ethnicity (White, Black, Hispanic and Others) and marital status (single, married, separated, divorced, or widowed). Tumor location (topography codes C710-C719) was also considered in this analysis [50], [51]. The relevant treatment variables included the extent of surgery (no surgery, local excision/biopsy, partial resection and gross total resection) [24], [50], [52] and radiation status (untreated and treated) [22], [24]. Since the cutoff of the last follow-up varied between SEER (December 31, 2013) and TCR (553 patients were followed up to May 2015), we set our cutoff date as December 31, 2013 in both SEER and TCR, which means we censored those who were still alive by December 31, 2013 in TCR. The primary endpoint was OS, which was defined as the subsequent months from diagnosis to the date of death due to any cause or the date of last follow-up or December 31, 2013. We also performed sub-analysis in TCR with extended follow-up up to May 2015, which could provide data for a longer term follow-up of OS associated with the usage of BEV in GBM patients.

### Ethics statement

Since SEER and TCR Limited-Use database are composed of existing and de-identified data, there is no individual patient-identifiable information. The study was approved by the Committee for the Protection of Human Subjects Committee from University of Texas Health Science Center at Houston (UTHealth), McGovern Medical School, Houston, TX.

### Statistical analysis

Descriptive statistics were used to depict and compare the cohort characteristics by calendar period of diagnosis using Mann-Whitney U test or Pearson's χ^2^ test. OS was assessed by applying Kaplan-Meier method and the difference between survival curves was tested by the two-sided log-rank test. Median OS, 1-year or 2-year survival rate (%) were calculated in patients who were observed for at least 1 or 2 years, and the difference in 1-year or 2-year survival rate across calendar period of diagnosis was tested by two-sided Chi-square test with Schouten correction. Univariate and multivariate analysis were applied by Cox proportional hazards model. In multivariate analysis, calendar period of diagnosis was included into models by adjusting the different sets of covariates: Model 1, Model 2 and Model 3 for SEER and TCR; Model 1, Model 2, Model 3 and Model 4 for the combined dataset (See details in Table 3, Table 4 and Table 5). The models were conducted to estimate HRs (95% CI) by adjusting for the potential confounding factors. After testing the proportional hazard assumption, the interactions between time and age at diagnosis, tumor site, surgery and radiation on OS were significant. Hence, stratified analysis Cox model was performed by involving the time-varying effect of age at diagnosis, tumor site, surgery and radiation. We obtained direct adjusted survival curves based on Model 3 for SEER, TCR, and Model 4 for the combined dataset, which computed the average of estimated survival curves for each patient, instead of generating adjusted curves by applying means of covariates [53], [54]. All statistical analyses were performed with Stata 12.0 (StataCorp, College Station, TX) and SAS (Version 9.3, SAS Institute, Cary, NC). *P* values were two-sided and considered statistically significant at *P* < 0.05.

## SUPPLEMENTARY MATERIALS FIGURES AND TABLES






