# Supplementary material for: Survival benefit of glioblastoma patients after FDA approval of temozolomide concomitant with radiation and bevacizumab: A population-based study
Source: Oncotarget. 2017 Apr 12;8(27):44015–31. doi: 10.18632/oncotarget.17054 (PMC5546458; doi:10.18632/oncotarget.17054)
Supplement: Supplementary file 1 [file oncotarget-08-44015-s001.pdf]

Survival benefit of glioblastoma patients after FDA approval of temozolomide concomitant with radiation and bevacizumab: A population-based study

SUPPLEMENTARY MATERIALS

SUPPLEMENTARY TABLES AND FIGURES

Supplementary Table 1: Comparsion of the related randomized clinical trials and population-based studies on TMZ and BEV in GBM treatment

See Supplementary File 1

Supplementary Table 2: Median OS, 1-year and 2-year survival rate by calendar period of diagnosis in SEER and TCR with extended follow-up*

See Supplementary File 2

Supplementary Table 3: Risk of death among GBM patients in relation to calendar period of diagnosis in TCR with extended follow-up (N=4355)

See Supplementary File 2

Supplementary Table 4: Risk of death in GBM patients by calendar period of diagnosis and by time-varying covariates in TCR with extended follow-up*

Time-varying covariates	Calendar Period of Diagnosis					
	P1		P2		P3	
	HR	95%CI	HR	95%CI	HR	95%CI
Age group at diagnosis						
20-49	1.00	-	0.85	0.70 - 1.02	0.55	0.45 - 0.68
50-59	1.00	-	0.80	0.69 - 0.93	0.52	0.44 - 0.60
60-69	1.00	-	0.83	0.71 - 0.96	0.61	0.52 - 0.70
70-	1.00	-	0.79	0.68 - 0.93	0.80	0.68 - 0.93
Tumor site						
Supratentorial	1.00	-	0.77	0.70 - 0.84	0.59	0.54 - 0.65
Infratentorial/ NOS	1.00	-	0.89	0.36 - 2.19	0.61	0.24 - 1.53
Surgery						
No surgery	1.00	-	0.91	0.75 - 1.10	0.81	0.67 - 0.98
Local excision/ biopsy	1.00	-	0.77	0.65 - 0.92	0.62	0.52 - 0.74
Partial resection	1.00	-	0.81	0.69 - 0.95	0.56	0.48 - 0.66
GTR	1.00	-	0.78	0.68 - 0.89	0.57	0.50 - 0.66
Radiation						
Untreated	1.00	-	0.85	0.72 - 1.00	0.64	0.55 - 0.75
Treated	1.00	-	0.78	0.71 - 0.86	0.63	0.57 - 0.69

Abbreviation: Calendar period of diagnosis, including P1, P2 and P3 (P1, Jan 2000-Feb 2005; P2, Mar 2005-Apr 2009; P3, May 2009-Dec 2013); NOS, not otherwise specified; GTR, gross total resection; HR, hazard ratio; 95%CI, 95% confidence interval.

*: Adjusted age at diagnosis, sex, race/ethnicity, marital status, tumor site, surgery and radiation as appropriate.

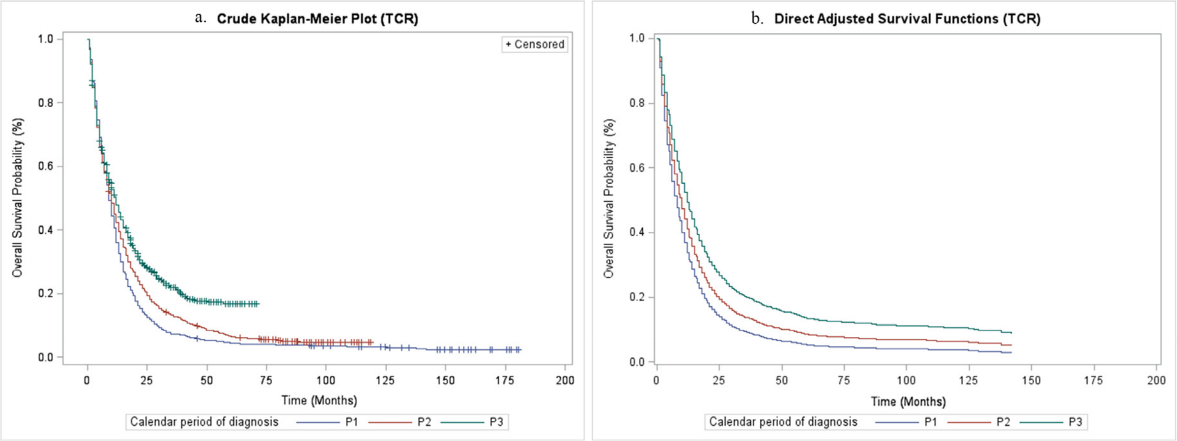

Supplementary Figure 1: Overall survival of GBM patients by calendar period of diagnosis in TCR with extended follow-up. **(a)** Crude overall survival in TCR with extended follow-up; **(b)** Direct adjusted survival after adjusting covariates in TCR with extended follow-up;

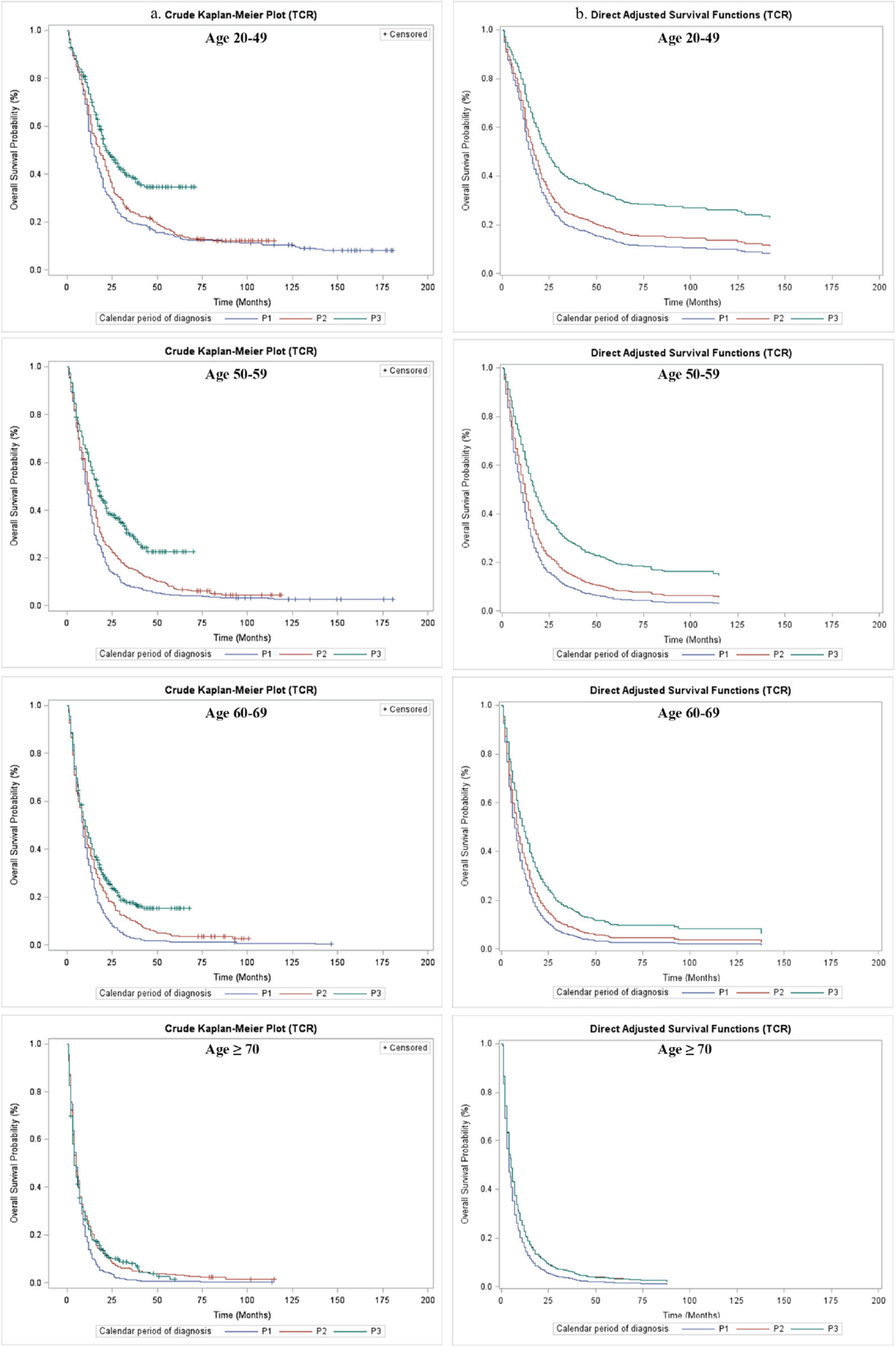

Supplementary Figure 2: Overall survival of GBM patients by calendar period of diagnosis and age group at diagnosis in TCR with extended follow-up. **(a)** Crude overall survival stratified by age group at diagnosis in TCR with extended follow-up; **(b)** Direct adjusted survival after adjusting covariates stratified by age group at diagnosis in TCR with extended follow-up;
