# Supplementary material for: Survival benefit of glioblastoma patients after FDA approval of temozolomide concomitant with radiation and bevacizumab: A population-based study
Source: Oncotarget. 2017 Apr 12;8(27):44015–31. doi: 10.18632/oncotarget.17054 (PMC5546458; doi:10.18632/oncotarget.17054)
Supplement: Supplementary file 3 [file oncotarget-08-44015-s003.doc]

| **Table S2**. Median OS, 1-year and 2-year Survival Rate by Calendar Period of Diagnosis in SEER and TCR with Extended Follow-up* | | | | | | | | | | |  |  |
| --- | --- | --- | --- | --- | --- | --- | --- | --- | --- | --- | --- | --- |
|  | Calendar Period of Diagnosis | | | | | | | | | | | |
| Survival statistics | Jan 2000 - Feb 2005 (P1) |  | Mar 2005 - Apr 2009 (P2) |  | May 2009 - Dec 2013 (P3) |  | Total  (P1, P2 and P3) |  | P1 vs P2 | P1 vs P3 | P2 vs P3 | Trend Test |
| **SEER** |  |  |  |  |  |  |  |  | *P^a^* | *P^b^* | *P^c^* | *P^d^* |
| Total cases, N | 8169 |  | 7420 |  | 8989 |  | 25478 |  | <0.001 | <0.001 | <0.001 | <0.001 |
| Death cases, N (%) | 7960 (97.4) |  | 7045 (94.9) |  | 6359 (70.7) |  | 13404 (54.5) |  |  |  |  |  |
| Median OS (months, IQR) | 8.0 (3.0-16.0) |  | 10.0 (4.0-20.0) |  | 11.0 (4.0-21.0) |  | 10.0 (4.0-19.0) |  |  |  |  |  |
| 1-year survival rate, % (95%CI) | 33.6 (32.6-34.7) |  | 42.1 (41.0-43.3) |  | 45.1 (43.9-46.2) |  | 40.0 (39.4-40.6) |  |  |  |  |  |
| 2-year survival rate, % (95%CI) | 12.6 (11.9-13.4) |  | 18.8 (17.9-19.7) |  | 19.8 (18.7-20.9) |  | 16.6 (16.1-17.1) |  |  |  |  |  |
| **TCR with extended follow-up**^e^ |  |  |  |  |  |  |  |  |  |  |  |  |
| Total cases, N | 1357 |  | 1286 |  | 1712 |  | 4355 |  | <0.001 | <0.001 | <0.001 | <0.001 |
| Death cases, N (%) | 1319 (97.2) |  | 1220 (94.9) |  | 1278 (74.6) |  | 3817 (87.6) |  |  |  |  |  |
| Median OS (months, IQR) | 9.0 (4.0-16.0) |  | 10.0 (4.0-20.9) |  | 12.0 (4.0-29.0) |  | 10.0 (4.0-21.0) |  |  |  |  |  |
| 1-year survival rate, % (95%CI) | 36.1 (33.6-38.7) |  | 42.5 (39.8-45.2) |  | 44.5 (41.8-47.1) |  | 41.0 (39.5-42.5) |  |  |  |  |  |
| 2-year survival rate, % (95%CI) | 13.3 (11.6-15.2) |  | 20.4 (18.3-22.7) |  | 19.4 (17.0-21.9) |  | 17.5 (16.3-18.8) |  |  |  |  |  |
| **SEER vs TCR with extended follow-up**^e^ |  |  |  |  |  |  |  |  |  |  |  |  |
| Log-rank test, *P* value | 0.247 |  | 0.677 |  | <0.001 |  | <0.001 |  |  |  |  |  |
| Abbreviation: OS, overall survival; IQR, interquartile range; 95%CI, 95% confidence interval. *: Using log-rank test tested the differences in Kaplan–Meier survival functions across calendar periods of diagnosis. The two-sided Chi-square test with Schouten correction in 1-year or 2-year survival rate between P1 and P2, P1 and P3, P2 and P3 in SEER or TCR were the same to Table 2 presented (not listed in Table S2), because 1-year or 2-year survival rate were calculated in patients who were observed for at least 1 or 2 years. Therefore, no difference in 1-year or 2-year survival rate was detected between TCR and TCR with extended follow-up. a: *P* value for comparison of survival functions between P1 and P2 in SEER or TCR with extended follow-up. b: *P* value for comparison of survival functions between P2 and P3 in SEER or TCR with extended follow-up. c: *P* value for comparison of survival functions between P1 and P3 in SEER or TCR with extended follow-up. d: Trend *P* value for survival functions across P1, P2 and P3 in SEER or TCR with extended follow-up.  e: TCR dataset with extended follow-up up to May, 2015. | | | | | | | | | | | | |

| **Table S3**. Risk of Death Among GBM Patients in Relation to Calendar Period of Diagnosis in TCR with Extended Follow-up (N=4355) | | | | | | | | | | | | | |
| --- | --- | --- | --- | --- | --- | --- | --- | --- | --- | --- | --- | --- | --- |
|  | Cases | Death | Age - sex adjusted | |  | Model 1^a^ | |  | Model 2^b^ | |  | Model 3^c^ | |
| Predictors | N | N | HR | 95%CI |  | HR | 95%CI |  | HR | 95%CI |  | HR | 95%CI |
| Calendar period of diagnosis |  |  |  |  |  |  |  |  |  |  |  |  |  |
| Jan 2000-Feb 2005 (P1) | 1357 | 1319 | 1.00 | - |  | 1.00 | - |  | 1.00 | - |  | 1.00 | - |
| Mar 2005-Apr 2009 (P2) | 1286 | 1220 | 0.82 | 0.76 - 0.88 |  | 0.82 | 0.76 - 0.89 |  | 0.82 | 0.76 - 0.89 |  | 0.80 | 0.74 - 0.87 |
| May 2009-Dec 2013 (P3) | 1712 | 1278 | 0.66 | 0.61 - 0.71 |  | 0.67 | 0.62 - 0.72 |  | 0.66 | 0.61 - 0.72 |  | 0.62 | 0.57 - 0.67 |
| Age at diagnosis |  |  |  |  |  |  |  |  |  |  |  |  |  |
| 20-49 | 801 | 622 | 1.00 | - |  | 1.00 | - |  | 1.00 | - |  | 1.00 | - |
| 50-59 | 1173 | 1001 | 1.44 | 1.30 - 1.59 |  | 1.43 | 1.29 - 1.58 |  | 1.42 | 1.29 - 1.58 |  | 1.39 | 1.26 - 1.54 |
| 60-69 | 1270 | 1133 | 1.91 | 1.73 - 2.11 |  | 1.89 | 1.71 - 2.09 |  | 1.89 | 1.70 - 2.08 |  | 1.84 | 1.66 - 2.03 |
| 70- | 1111 | 1061 | 3.16 | 2.85 - 3.50 |  | 3.10 | 2.79 - 3.44 |  | 3.11 | 2.81 - 3.45 |  | 2.87 | 2.58 - 3.18 |
| Sex |  |  |  |  |  |  |  |  |  |  |  |  |  |
| Male | 2607 | 2285 | 1.00 | - |  | 1.00 | - |  | 1.00 | - |  | 1.00 | - |
| Female | 1748 | 1532 | 1.02 | 0.95 - 1.08 |  | 1.00 | 0.94 - 1.07 |  | 1.00 | 0.94 - 1.07 |  | 0.99 | 0.92 - 1.06 |
| Race/Ethnicity |  |  |  |  |  |  |  |  |  |  |  |  |  |
| White | 3274 | 2927 |  |  |  | 1.00 | - |  | 1.00 | - |  | 1.00 | - |
| Black | 269 | 232 |  |  |  | 1.03 | 0.90 - 1.18 |  | 1.02 | 0.90 - 1.17 |  | 0.98 | 0.86 - 1.12 |
| Hispanic | 715 | 587 |  |  |  | 0.91 | 0.83 - 1.00 |  | 0.90 | 0.82 - 0.98 |  | 0.87 | 0.79 - 0.95 |
| Others | 97 | 71 |  |  |  | 0.77 | 0.61 - 0.97 |  | 0.76 | 0.60 - 0.96 |  | 0.72 | 0.56 - 0.91 |
| Marital status |  |  |  |  |  |  |  |  |  |  |  |  |  |
| Single | 609 | 501 |  |  |  | 1.00 | - |  | 1.00 | - |  | 1.00 | - |
| Married | 3022 | 2666 |  |  |  | 0.97 | 0.88 - 1.07 |  | 0.98 | 0.89 - 1.08 |  | 1.02 | 0.93 - 1.13 |
| DWS | 724 | 650 |  |  |  | 1.09 | 0.96 - 1.23 |  | 1.10 | 0.97 - 1.24 |  | 1.13 | 1.00 - 1.28 |
| Tumor site |  |  |  |  |  |  |  |  |  |  |  |  |  |
| Supratentorial | 3423 | 2988 |  |  |  |  |  |  | 1.00 | - |  | 1.00 | - |
| Infratentorial/NOS | 932 | 829 |  |  |  |  |  |  | 1.25 | 1.16 - 1.36 |  | 1.16 | 1.07 - 1.25 |
| Surgery |  |  |  |  |  |  |  |  |  |  |  |  |  |
| No surgery | 753 | 697 |  |  |  |  |  |  |  |  |  | 1.00 | - |
| Local excision/biopsy | 877 | 789 |  |  |  |  |  |  |  |  |  | 0.69 | 0.63 - 0.77 |
| Partial resection | 1164 | 1014 |  |  |  |  |  |  |  |  |  | 0.75 | 0.68 - 0.82 |
| GTR | 1561 | 1317 |  |  |  |  |  |  |  |  |  | 0.57 | 0.52 - 0.63 |
| Radiation |  |  |  |  |  |  |  |  |  |  |  |  |  |
| Untreated | 1332 | 1167 |  |  |  |  |  |  |  |  |  | 1.00 | - |
| Treated | 3023 | 2650 |  |  |  |  |  |  |  |  |  | 0.72 | 0.67 - 0.77 |
| Abbreviation: DWS, divorced or widowed or separated; NOS, not otherwise specified; GTR, gross total resection; HR, hazard ratio; 95%CI, 95% confidence interval. | | | | | | | | | | | | | |
| a: Model 1: Adjusted age at diagnosis, sex, race/ethnicity and marital status. | | | | | | | | | | | | | |
| b: Model 2: Adjusted age at diagnosis, sex, race/ethnicity, marital status and tumor site. | | | | | | | | | | | | | |
| c: Model 3: Adjusted age at diagnosis, sex, race/ethnicity, marital status, tumor site, surgery and radiation. | | | | | | | | | | | | | |
